# Factors Associated With Pain Interference Among Black and White Older Adults

**DOI:** 10.1093/geroni/igaa057.1389

**Published:** 2020-12-16

**Authors:** Dottington Fullwood, Roger Fillingim, Alisa Johnson, Nancy Gell

**Affiliations:** 1 The University of Florida, Gainesville, Florida, United States; 2 University of Florida, Pain Research and Intervention Center of Excellence, Gainesville, Florida, United States; 3 University of Florida, Gainesville, Florida, United States; 4 The University of Vermont, Burlington, Vermont, United States

## Abstract

Pain interference (PI) is an indicator of pain impact and is associated with physical performance (PP). However, factors associated with PI among older adults are not well described, including associations with PP and racial differences. This study explored PI among older adults by race. Data were obtained from the 2013 Pain Supplement of the National Health and Aging Trends Study (N= 1,202; 59.9% female, 23.0% Black non-Hispanic). Interviews included questions on sociodemographics, multi-morbidities, pain intensity (0-10 scale), and PI overall. Participants were also asked “In the last month, how much did pain interfere with ADLs, household activities, going outside, shopping, social activities and walking, which was used to create a PI index (Range 0-18). Physical performance measures assessed balance, gait speed, and chair stands (Short physical performance battery; SPPB). Logistic and multivariable regression analyses were conducted to determine associations among PI with PP, pain intensity, and race. Older Black adults experienced higher pain intensity (3.90 vs. 3.03) and demonstrated greater PP limitations (5.4 vs. 7.1 SPPB score) compared to older White adults (p<0.001). Higher scores on the PI index were associated with worse PP, higher pain intensity, depression, multi-morbidity, and White race (p <0.001), independent of confounders. Similarly, more PI overall was associated with White race, higher pain intensity, worse PP, and multi-morbidity (p<0.001). Despite higher pain intensity and worse physical performance, older Black adults reported lower PI than White older adults. Additional exploration is needed to discern the paradoxically lower PI among older Black adults, including potential resilience factors.

